# Supplementary material for: Computational models applied to metabolomics data hints at the relevance of glutamine metabolism in breast cancer
Source: BMC Cancer. 2020 Apr 15;20:307. doi: 10.1186/s12885-020-06764-x (PMC7265650; doi:10.1186/s12885-020-06764-x)
Supplement: Supplementary file 5 — Table S2: Multivariate Cox regression model comparing OS predictor based on metabolomics data. T = tumor stage, N = lymph node status, G = tumor grade. [file 12885_2020_6764_MOESM5_ESM.docx]

| Multivariate analysis | p-value |
| --- | --- |
| T | 0.863 |
| N | 0.014 |
| G | 0.246 |
| Predictor metabolites | 0.018 |

Sup Table 2: Multivariate Cox regression model comparing OS predictor based on metabolomics data. T = tumor stage, N = lymph node status, G = tumor grade.
