# Supplementary material for: Computational models applied to metabolomics data hints at the relevance of glutamine metabolism in breast cancer
Source: BMC Cancer. 2020 Apr 15;20:307. doi: 10.1186/s12885-020-06764-x (PMC7265650; doi:10.1186/s12885-020-06764-x)
Supplement: Supplementary file 6 — Table S3: Multivariate Cox regression model comparing OS predictor based on node activity of lipid metabolism. T = tumor stage, N = lymph node status, G = tumor grade. [file 12885_2020_6764_MOESM6_ESM.docx]

| Multivariate analysis | p-value |
| --- | --- |
| T | 0.732 |
| N | 0.030 |
| G | 0.464 |
| Predictor node activity of lipid metabolism | 0.141 |

Sup Table 3: Multivariate Cox analysis comparing predictor based on node activity of lipid metabolism. T = tumor stage, N = lymph node status, G = tumor grade.
