# Supplementary material for: Computational models applied to metabolomics data hints at the relevance of glutamine metabolism in breast cancer
Source: BMC Cancer. 2020 Apr 15;20:307. doi: 10.1186/s12885-020-06764-x (PMC7265650; doi:10.1186/s12885-020-06764-x)
Supplement: Supplementary file 7 — Table S4: Multivariate Cox regression model comparing OS predictor based on flux activities. T = tumor stage, N = lymph node status, G = tumor grade. [file 12885_2020_6764_MOESM7_ESM.docx]

| Multivariate analysis | p-value |
| --- | --- |
| T | 0.489 |
| N | 0.058 |
| G | 0.351 |
| Predictor flux activities | 0.028 |

Sup Table 4: Multivariate Cox regression comparing predictor based on flux activities. T = tumor stage, N = lymph node status, G = tumor grade.
