# Supplementary material for: Computational models applied to metabolomics data hints at the relevance of glutamine metabolism in breast cancer
Source: BMC Cancer. 2020 Apr 15;20:307. doi: 10.1186/s12885-020-06764-x (PMC7265650; doi:10.1186/s12885-020-06764-x)
Supplement: Supplementary file 8 — Table S5: Metabolites associated with flux activity of each network branch. [file 12885_2020_6764_MOESM8_ESM.docx]

| Network Branch | Flux activity | Number of metabolites in this branch related to the flux activity | Name of metabolites in this branch related to the flux activity | |
| --- | --- | --- | --- | --- |
| Branch 1 | Glycolysis/gluconeogenesis | 3 | citrate, fructose 6-P, 3-phosphoglycerate | |
| Branch 1 | Pyrimidine synthesis | 2 | guanosine, inosine | |
| Branch 2 | Androgen and estrogen metabolism | 0 |  | |
| Branch 2 | ROS detoxification | 0 | |  |
| Branch 2 | Sphingolipid metabolism | 0 |  | |
| Branch 2 | Vitamin A metabolism | Not quantifiable |  | |
| Branch 2 | Vitamin B6 metabolism | At least 1 | glutamate | |
| Branch 2 | Vitamin D metabolism | Not quantifiable |  | |
| Branch 3 | Glycine, serine, alanine and threonine metabolism | 5 | methionine, pyruvate, glycine, asparagine, threonine | |
| Branch 3 | D-alanine metabolism | 5 | methionine, pyruvate, glycine, asparagine, threonine | |
| Branch 3 | Fructose and mannose metabolism | 1 | pyruvate | |
| Branch 3 | Glutathione metabolism | 2 | pyruvate, glycine | |
| Branch 3 | Nucleotide salvage pathway | 2 | pyruvate, glycine | |
| Branch 3 | Phenylalanine metabolism | 6 | pyruvate, valine, isoleucine, tyrosine, leucine, histidine | |
| Branch 3 | Purine catabolism | 2 | pyruvate, glycine | |
| Branch 3 | Tetrahydrobiopterin metabolism | 1 | tyrosine | |
| Branch 3 | Triacylglycerol synthesis | 0 |  | |
| Branch 3 | Vitamin C metabolism | Not quantifiable |  | |
| Branch 3 | Nucleotide interconversion | 2 | pyruvate, glycine | |
| Branch 4 | Alanine and aspartate metabolism | 4 | choline, glycerate, citrulline, urea | |
| Branch 4 | Butanoate metabolism | 1 | 2-hydroxybutyrate | |
| Branch 4 | Cholesterol metabolism | 0 |  | |
| Branch 4 | Citric acid cycle | 0 |  | |
| Branch 4 | Coenzyme A catabolism | 1 | 2-hydroxystearate | |
| Branch 4 | Coenzyme A synthesis | 0 |  | |
| Branch 4 | Galactose metabolism | 0 |  | |
| Branch 4 | Glutamate metabolism | 1 | dimethylarginine | |
| Branch 4 | Nicotinamide adenine dinucleotide metabolism | 1 | 1-methylnicotinamide | |
| Branch 4 | Oxidative phosphorylation | 1 | citrulline | |
| Branch 4 | Pentose phosphate pathway | 1 | glycerate | |
| Branch 4 | Propanoate metabolism | 2 | 2-propanediol, 2-hydroxybutyrate | |
| Branch 4 | Pyruvate metabolism | 1 | 2-propanediol | |
| Branch 4 | Taurine and hypotaurine metabolism | 0 |  | |
| Branch 4 | Tryptophan metabolism | 0 |  | |
| Branch 4 | Urea cycle | 3 | urea, citrulline, creatinine | |
| Branch 4 | beta-Alanine metabolism | 5 | 6-dihydrouracil, citrulline, urea, glycerate, choline | |
| Branch 5 | Glyoxylate and dicarboxylate metabolism | 1 | phosphate | |
| Branch 5 | Vitamin B2 metabolism | 1 | phosphate | |
| Branch 6 | Valine, leucine and isoleucine metabolism | 0 |  | |
| Branch 6 | Lysine metabolism | 1 | S-adenosylhomocysteine | |
| Branch 6 | Histidine metabolism | 3 | S-adenosylhomocysteine, histamine, heme | |
| Branch 6 | Arginine and proline metabolism | 3 | S-adenosylhomocysteine, heme, spermidine | |
| Branch 6 | Arachidonic acid metabolism | 1 | heme | |
| Branch 7 | Aminosugar metabolism | 1 | N-acetylneuraminate | |
| Branch 7 | Eicosanoid metabolism | 3 | eicosapentaenoate, arachidonate, glycerol | |
| Branch 7 | Folate metabolism | 1 | glucose | |
| Branch 7 | Glycerophospholipid metabolism | 11 | margarate, oleate, stearate, linolenate, arachidonate, palmitate, linoleate, cis-vaccenate, eicosapentaenoate, docosahexaenoate, glycerol | |
| Branch 7 | Hyaluronan metabolism | 10 | margarate, oleate, stearate, linolenate, arachidonate, palmitate, linoleate, cis-vaccenate, eicosapentaenoate, docosahexaenoate | |
| Branch 7 | Methionine and cysteine metabolism | 0 |  | |
| Branch 7 | Phosphatidylinositol phosphate metabolism | 1 | glycerol | |
| Branch 7 | Pyrimidine catabolism | 1 | ornithine | |
| Branch 7 | Starch and sucrose metabolism | 1 | glucose | |
| Branch 7 | Steroid metabolism | 2 | arachidonate, palmitate | |
| Branch 7 | Tyrosine metabolism | 1 | succinate | |

Sup Table 5: Metabolites associated with flux activity of each network branch.
