# Supplementary material for: Computational models applied to metabolomics data hints at the relevance of glutamine metabolism in breast cancer
Source: BMC Cancer. 2020 Apr 15;20:307. doi: 10.1186/s12885-020-06764-x (PMC7265650; doi:10.1186/s12885-020-06764-x)
Supplement: Supplementary file 9 — Fig S1: Node activities from the metabolic network. Fig S2: Tumor growth rate predicted using FBA for ER+ and ER- tumors. Fig S3: Flux activities were significantly different between ER+ and ER-. -. ****, ≤ 0.0001; ***, ≤ 0.001; ** , ≤ 0.01 ; * ≤ 0.05 [file 12885_2020_6764_MOESM9_ESM.docx]

Sup Fig 1





Sup Fig 2





Sup Fig 3
